# Notch Signaling Inhibition by LY411575 Attenuates Osteoblast Differentiation and Decreased Ectopic Bone Formation Capacity of Human Skeletal (Mesenchymal) Stem Cells

**DOI:** 10.1155/2019/3041262

**Published:** 2019-08-22

**Authors:** Nihal AlMuraikhi, Dalia Ali, Radhakrishnan Vishnubalaji, Muthurangan Manikandan, Muhammad Atteya, Abdulaziz Siyal, Musaad Alfayez, Abdullah Aldahmash, Moustapha Kassem, Nehad M. Alajez

**Affiliations:** ^1^Stem Cell Unit, Department of Anatomy, College of Medicine, King Saud University, Riyadh 11461, Saudi Arabia; ^2^Molecular Endocrinology Unit (KMEB), Department of Endocrinology, University Hospital of Odense and University of Southern Denmark, Odense, Denmark; ^3^Cancer Research Center, Qatar Biomedical Research Institute (QBRI), Hamad Bin Khalifa University (HBKU), Qatar Foundation (QF), PO Box 34110, Doha, Qatar; ^4^Histology Department, Faculty of Medicine, Cairo University, Cairo, Egypt; ^5^Prince Naif Health Research Center, King Saud University, Riyadh 11461, Saudi Arabia; ^6^Department of Cellular and Molecular Medicine, Danish Stem Cell Center (DanStem), University of Copenhagen, 2200 Copenhagen, Denmark

## Abstract

**Background:**

Chemical biology approaches using small molecule inhibitors targeting specific signaling pathways are useful tools to dissect the molecular mechanisms governing stem cell differentiation and for their possible use in therapeutic interventions.

**Methods:**

Stem cell signaling small molecule library functional screen was performed employing human bone marrow skeletal (mesenchymal) stem cells (hBMSCs). Alkaline phosphatase (ALP) activity and formation of mineralized matrix visualized by Alizarin red staining were employed as markers for osteoblastic differentiation. Global gene expression profiling was conducted using the Agilent microarray platform, and data normalization and bioinformatics were performed using GeneSpring software. Pathway analyses were conducted using the Ingenuity Pathway Analysis (IPA) tool. *In vivo* ectopic bone formation was performed using hBMSC mixed with hydroxyapatite–tricalcium phosphate granules that were implanted subcutaneously in 8-week-old female nude mice. Hematoxylin and eosin staining and Sirius red staining were performed to identify bone formation *in vivo*.

**Results:**

Among the tested molecules, LY411575, a potent *γ*-secretase and Notch signaling inhibitor, exhibited significant inhibitory effects on osteoblastic differentiation of hBMSCs manifested by reduced ALP activity, mineralized matrix formation, and decreased osteoblast-specific gene expression as well as *in vivo* ectopic bone formation. Global gene expression profiling of LY411575-treated cells revealed changes in multiple signaling pathways, including focal adhesion, insulin, TGF*β*, IL6, and Notch signaling, and decreased the expression of genes associated with functional categories of tissue development. Among the affected signaling networks were TGF*β*1, SPP1, and ERK regulatory networks.

**Conclusions:**

We identified *γ*-secretase inhibitor (LY411575) as a potent regulator of osteoblastic differentiation of hBMSC that may be useful as a therapeutic option for treating conditions associated with ectopic bone formation.

## 1. Introduction

Human bone marrow skeletal (mesenchymal) stem cells (hBMSC) are clonogenic cells present within bone marrow stroma and capable of differentiation to various mesoderm-type cells including osteoblasts and adipocytes [1]. The osteoblastic differentiation fate of hBMSCs is tightly regulated by intracellular signaling pathways [2] that include Notch [3], TGF*β* [4], bone morphogenetic proteins [3], and Wnt/*β*-catenin [5].

In mammals, the Notch family has four receptors, Notch1-4, and five receptor-binding ligands, Jagged1/2 and delta-like 1/3/4 [6]. Notch signaling is activated when a cognate ligand binds to Notch receptor leading to a sequential proteolytic cleavage by *γ*-secretase enzyme and the release of the Notch intracellular domain (NICD) [7]. NICD is the functional part of Notch signaling which translocates into the nucleus and regulates target gene transcription [3, 7]. In addition to Notch, *γ*-secretase enzyme is a vital proteolytic enzyme that catalyzes the cleavage of additional integral membrane proteins such as Amyloid Beta Precursor Protein (APP), CD44, and N-cadherin [8]. Previous studies have demonstrated that the Notch signaling pathway is a regulator of bone formation [3, 9, 10]. Notch signaling has been reported either to enhance or to inhibit osteoblast differentiation and mineralization which is dependent on the cell model utilized and culture conditions [9].

Chemical biology approaches employing small molecule inhibitors are useful tools to target specific intracellular signaling pathways in order to dissect mechanisms involved in stem cell differentiation [11, 12]. Here, we identified a small molecule LY411575 through a small molecule library screen [11], as an inhibitor of in vitro osteoblast differentiation and in vivo ectopic bone formation of hBMSC. Global gene expression analysis revealed that LY411575 affects, in addition to Notch target genes, a number of intracellular signaling networks known to influence osteoblast differentiation and bone formation.

## 2. Materials and Methods

### 2.1. Cell Culture

A telomerized hBMSC line (hBMSC-TERT) was used in this study as a model for primary hBMSC. hBMSC-TERT was created through an overexpression of the human telomerase reverse transcriptase (hTERT) gene. hBMSC-TERT expresses typical characteristics of primary hBMSC including stemness markers, multipotency, and molecular signature of global gene expression [13–16].

The cells were cultured in DMEM, a basal medium supplemented with 4,500 mg/l D-glucose, 4 mM L-glutamine, and 110 mg/l 10% sodium pyruvate, in addition to 10% fetal bovine serum (FBS), 1% penicillin-streptomycin, and 1% nonessential amino acids. All reagents were purchased from Thermo Fisher Scientific Life Sciences, Waltham, MA (http://www.thermofisher.com). Cells were incubated in 5% CO_2_ incubators at 37°C and 95% humidity.

### 2.2. Osteoblastic Differentiation

At 80%–90% confluence, the cells were incubated in osteoblast induction medium (DMEM) containing 10% FBS, 1% penicillin-streptomycin, 50 mg/ml L-ascorbic acid (Wako Chemicals GmbH, Neuss, Germany, http://www.wako-chemicals.de/), 10 mM b-glycerophosphate (Sigma-Aldrich), 10 nM calcitriol (1a,25-dihydroxyvitamin D3; Sigma-Aldrich), and 10 nM dexamethasone (Sigma-Aldrich). The stem cell signaling small molecule inhibitor library and LY411575 were purchased from Selleckchem Inc. (Houston, TX, http://www.selleckchem.com). Small molecule inhibitors were added to the osteoblast induction medium at a concentration of 3.0 *μ*M. The cells were exposed to the inhibitor or vehicle dimethyl sulfoxide (DMSO) throughout the differentiation period.

### 2.3. Cell Viability Assay

Cell viability assay was done using the alamarBlue assay according to the manufacturer's recommendations (Thermo Fisher Scientific) where cells were cultured in 96-well plates in 200 *μ*l of the medium. On day10, 20 *μ*l/well (10%) of alamarBlue substrate was added and plates were incubated for 1 hr in the dark at 37°C. Readings were taken using BioTek Synergy II microplate reader (BioTek Inc., Winooski, VT, US) using fluorescent mode (Ex 530 nm/Em 590 nm).

### 2.4. Quantification of Alkaline Phosphatase Activity

Alkaline phosphatase (ALP) activity quantification was performed using the BioVision ALP activity colorimetric assay kit (BioVision Inc., Milpitas, CA, http://www.biovision.com/) with some adjustments. The cells were cultured in 96-well plates for 10 days. The cells were then rinsed once with PBS and fixed using 3.7% formaldehyde in 90% ethanol for 30 seconds at room temperature. Fixative was removed, and 50 *μ*l/well of p-nitrophenyl phosphate solution was added and incubated for 30–60 minutes. Using a SpectraMax/M5 fluorescence spectrophotometer plate reader, the optical densities were then measured at 405 nm and ALP enzymatic activity was normalized to cell number.

### 2.5. Alkaline Phosphatase Staining

Alkaline phosphatase staining was performed at day 10 of osteoblast differentiation. Cultured cells were washed in PBS and fixed in 10 mM acetone/citrate buffer at pH 4.2 for 5 min at room temperature. The fixative was replaced with Naphthol/Fast Red stain (0.2 mg/ml of Naphthol AS-TR phosphate substrate (Sigma); 0.417 mg/ml of Fast Red (Sigma)) for 1 hr at room temperature. Then, cells were washed with water and scanned under the microscope.

### 2.6. Alizarin Red S Staining for Mineralized Matrix Formation

Alizarin red S staining was performed on day 21 of osteoblast differentiation. Cultured cells were washed twice with PBS and fixed with 4% paraformaldehyde for 15 min at room temperature. The fixative was removed, and the cells were then rinsed with distilled water and stained with the 2% Alizarin red S staining kit (ScienceCell Research Laboratories, Cat. No. 0223) for 20–30 min at room temperature. Subsequently, the cells were washed with water and scanned under the microscope.

### 2.7. RNA Extraction and cDNA Synthesis

Total RNA was extracted from cell pellets at day 10 and 21 postosteoblast differentiation induction, using the Total RNA Purification Kit (Norgen Biotek Corp., Thorold, ON, Canada, https://norgenbiotek.com/) according to the manufacturer's instructions. The concentrations of total RNA extracted were measured using NanoDrop 2000 (Thermo Fisher Scientific Life Sciences). cDNA was synthesized using 500 ng of total RNA using the cDNA Transcription Kit (Thermo Fisher Scientific Life Sciences) according to the manufacturer's instructions.

Quantitative reverse transcriptase-polymerase chain reaction (qRT-PCR) was performed using the Applied Biosystems ViiA™ 7 Real-Time PCR System (Thermo Fisher Scientific Life Sciences). Primers used in the presented study are listed in Table 1. Relative expression was calculated using the 2∆CT value method, and analysis was performed as previously described [17].

### 2.8. Gene Expression Profiling and Pathway Analyses

One hundred fifty nanograms of total RNA from day 10 osteoblastic differentiated hBMSCs were labeled using a low input Quick Amp Labeling Kit (Agilent Technologies, Santa Clara, CA, http://www.agilent.com) and hybridized to the Agilent Human SurePrint G3 Human GE 8 × 60 k microarray chip. All microarray experiments were accomplished at the Microarray Core Facility (Stem Cell Unit, Department of Anatomy, King Saud University College of Medicine, Riyadh, Saudi Arabia). The resulted data were normalized and analyzed using GeneSpring 13.0 software (Agilent Technologies). Pathway analysis was performed using GeneSpring 13.0 as described previously [18]. Twofold cutoff and *P*(corr) < 0.05 (Benjamini-Hochberg multiple testing corrected) were used to define significantly changed transcripts. Pathway and functional annotation analysis were conducted using the Ingenuity pathway (Ingenuity Systems, http://www.ingenuity.com) [19]. Differentially expressed genes exhibiting ≥2 FC (fold change) and corrected *P* value < 0.05 were chosen for analysis. Enriched network categories were algorithmically generated based on their connectivity and ranked according to *Z* score.

### 2.9. *In Vivo* Ectopic Bone Formation Assay

Ethical approval for all animal experiments was granted by the Animal Care Committees of King Saud University (No. KSU-SE-18-2). All in vivo experiments were conducted as per the guidelines of the Animal Care Committees of King Saud University. In vivo experiments were performed as previously described [11, 20]. Briefly, the cells were trypsinized to a single-cell suspension and resuspended in a culture medium with/without the tested drug, LY411575 (3.0 *μ*M). Around 5 × 10^5^ cells were seeded onto 40 mg Triosite hydroxyapatite–tricalcium phosphate granules per implant (HA/TCP, Biomatlante/Zimmer, Albertslund, Denmark; 0.5 to 1 mm granules) and kept overnight at 37°C, 5% CO_2_. HA/TCP granules in combination with cells were then implanted subcutaneously (four implants per cell line) in the dorsolateral area of immune-compromised nude mice for 4 weeks. The implants were recovered, fixed in formalin, decalcified using formic acid solution (0.4 M formic acid and 0.5 M sodium formate), for three days, embedded, and sectioned at 4 *μ*m. Staining was performed with hematoxylin and eosin or Sirius red to identify the areas of newly formed bone.

### 2.10. Quantification of Ectopic Bone Formation

Slides were digitized using high-resolution whole-slide digital ScanScope scanner (Aperio Technologies Inc.). The digital images from hematoxylin and eosin-stained slides were then viewed and quantified using the tools of ImageJ software. The whole implant is contoured to obtain the total implant area in pixels (TA). All areas of bone are selected to get a total bone area in pixels (BA). The BA/TA ratio was calculated and reported as percentage (*n* = 3 sections per implant and 4 implants/treatment). The digital images from Sirius red-stained slides were viewed and analyzed using Aperio's viewing and image analysis tools. In each slide, five rectangular fields of a fixed area of 1.18 mm^2^ were randomly selected. Color deconvolution (color separation) algorithm (Aperio Technologies Inc.) was then applied so as to detect and measure the area of red color of stained collagen and calculate its area relative to the total area.

### 2.11. Statistical Analysis

Statistical analysis and graphing were performed using Microsoft Excel 2016 and GraphPad Prism 8 software (GraphPad software, San Diego, CA, U.S.A.), respectively. Results were presented as mean ± SEM from at least two independent experiments, and statistical testing was performed using the unpaired, two-tailed Student *t*-test. *P* values < 0.05 were considered statistically significant.

## 3. Results

### 3.1. LY411575 Inhibits Osteoblast Differentiation of hBMSCs

**Figure 1:**
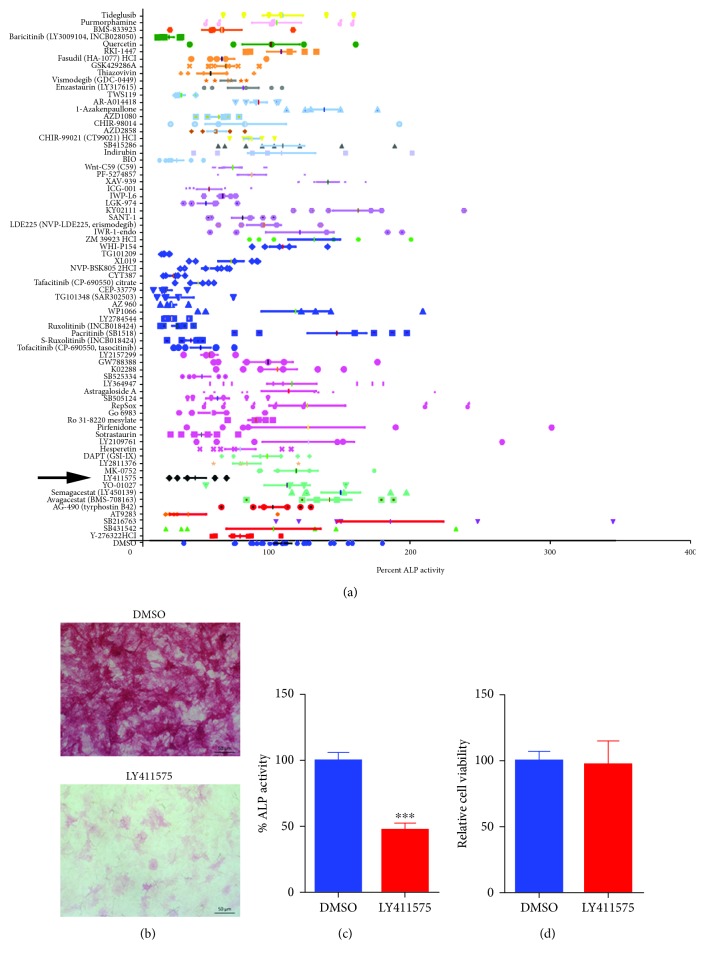
Effects of LY411575 on the osteoblast differentiation of hBMSCs. (a) Scatter plot depicting the initial ALP functional screen of 73 compounds for their effects on day 10 postosteoblastic differentiation of hBMSCs. *Y*-axis indicates the small molecule inhibitor, while each symbol represents replica. *X*-axis indicates percent change in ALP activity compared to DMSO control-treated cells. (b) Representative alkaline phosphatase (ALP) staining of hBMSCs on day10 postosteoblastic differentiation, in the presence of LY411575 (3.0 *μ*M) compared to DMSO-treated control cells. Images 10x magnification. Abbreviations: ALP: alkaline phosphatase; DMSO: dimethyl sulfoxide. (c) Quantification of ALP activity in hBMSCs following treatment with LY411575 (3.0 *μ*M) versus DMSO vehicle-treated control cells on day 10 osteoblast differentiation. Data are presented as mean percentage ALP activity ± SEM (*n* = 16). (d) Assay for cell number employing alamarBlue assay following treatment with LY411575 (3.0 *μ*M) versus DMSO vehicle-treated control cells on day 10 postosteoblastic differentiation.

We have recently reported the result of a small molecule library screen that identified several inhibitors of osteoblast differentiation of hBMSCs [11]. Among these, LY411575 exhibited potent inhibitory effects (Figure 1(a)). hBMSCs treated with LY411575 (3 *μ*M) exhibited marked reduction in ALP cytochemical staining and ALP activity compared to DMSO-vehicle-treated control cells (Figures 1(b) and 1(c)). LY411575 did not affect hBMSC cell viability, implying a specific impairment of osteoblastic differentiation (Figure 1(d)).

**Figure 2:**
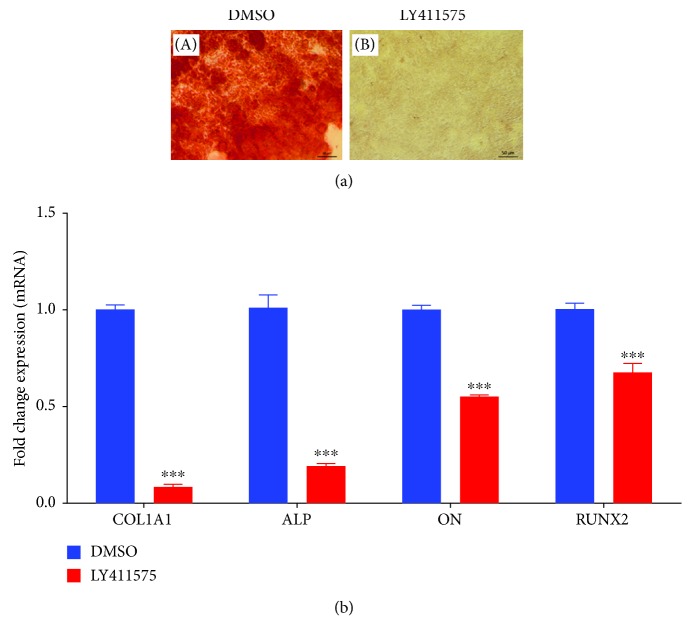
LY411575 inhibits mineralized matrix formation of hBMSCs. (a) Cytochemical staining for mineralized matrix formation using Alizarin red stain performed on day 21 postosteoblastic differentiation in the absence (A) or presence (B) of LY411575 (3.0 *μ*M). Magnification 10x. (b) Quantitative RT-PCR analysis for the gene expression of ALP, COL1A1, ON, and RUNX2 in hBMSCs on day 21 postosteoblast differentiation in the absence (blue) or presence (red) of LY411575 (3.0 *μ*M). Gene expression was normalized to *β*-actin. Data are presented as mean fold change ± SEM (*n* = 6). ^∗∗∗^*P* ≤ 0.005. Abbreviations: ALP: alkaline phosphatase; COL1A1: collagen type I alpha 1; ON: osteonectin; RUNX2: runt-related transcription factor 2; DMSO: dimethyl sulfoxide.

hBMSCs treated with LY411575 (3 *μ*M) revealed substantial reduction in mineralized matrix formation that stained positive for Alizarin red, in LY411575-treated hBMSCs compared to vehicle-treated controls (Figure 2(a)). In addition, LY411575 reduced the expression of several osteoblast-specific gene marker, namely, ALP, COL1A1, ON, and RUNX2 measured on day 21 postinduction of osteoblast differentiation (Figure 2(b)).

### 3.2. Global Transcriptome Analysis Revealed Multiple Altered Signaling Pathways in Response to LY411575 Treatment

**Figure 3:**
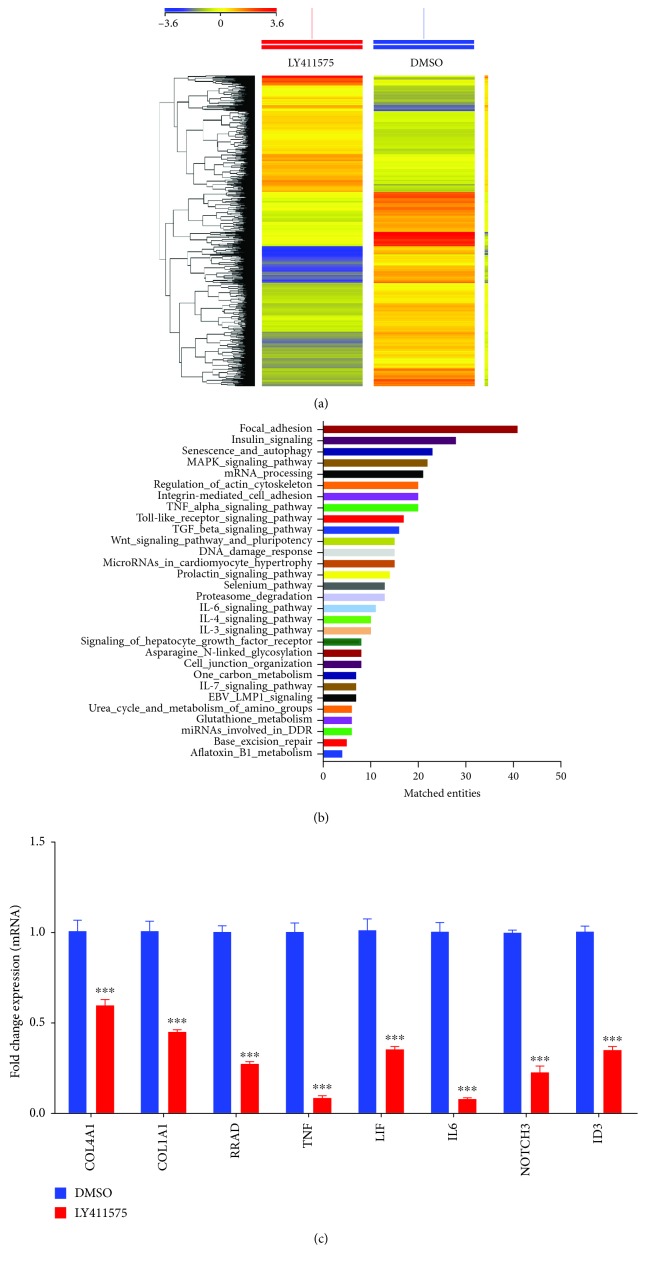
LY411575 affects multiple genetic pathways in hBMSCs. (a) Heat map and unsupervised hierarchical clustering performed on differentially expressed genes during osteoblastic differentiation of LY411575-treated hBMSCs compared to DMSO-treated control cells. (b) Bar chart illustrating the distribution of the top twenty enriched genetic pathways enriched in the significantly downregulated genes identified in LY411575-treated hBMSCs compared to DMSO-treated control cells. (c) Validation of a selected panel of downregulated genes in LY411575-treated hBMSCs compared to DMSO-treated control using qRT-PCR. Gene expression was normalized to *β*-actin. Data are presented as mean fold change ± SEM (*n* = 6); ^∗∗∗^*P* < 0.001.

To define the molecular mechanisms by which LY411575 inhibits osteoblastic differentiation of hBMSCs, we performed a global gene expression profiling followed by bioinformatic analysis of LY411575-treated hBMSC as compared to vehicle-treated control cells. Heat map revealed large number of differentially expressed genes in LY411575-treated cells compared to DMSO-treated control cells (Figure 3(a)), and we observed the presence of 1432 upregulated and 2379 downregulated genes (fold change ≥2.0; *P*(corr) < 0.05) (Supplementary [Supplementary-material supplementary-material-1]). Pathway analysis of the downregulated genes identified several differentially regulated signaling pathways known to be associated with osteoblast differentiation and functions, e.g., Notch, focal adhesion, insulin, TGF*β*, and IL6 signaling (Figure 3(b)). To validate the results of microarray, we selected a number of genes: COL1A1, COL4A1, RRAD, TNF, LIF, IL6, NOTCH3, and ID3, and we tested the changes in their expression using qRT-PCR. The results were collectively in agreement with the microarray data (Figure 3(c)).

**Figure 4:**
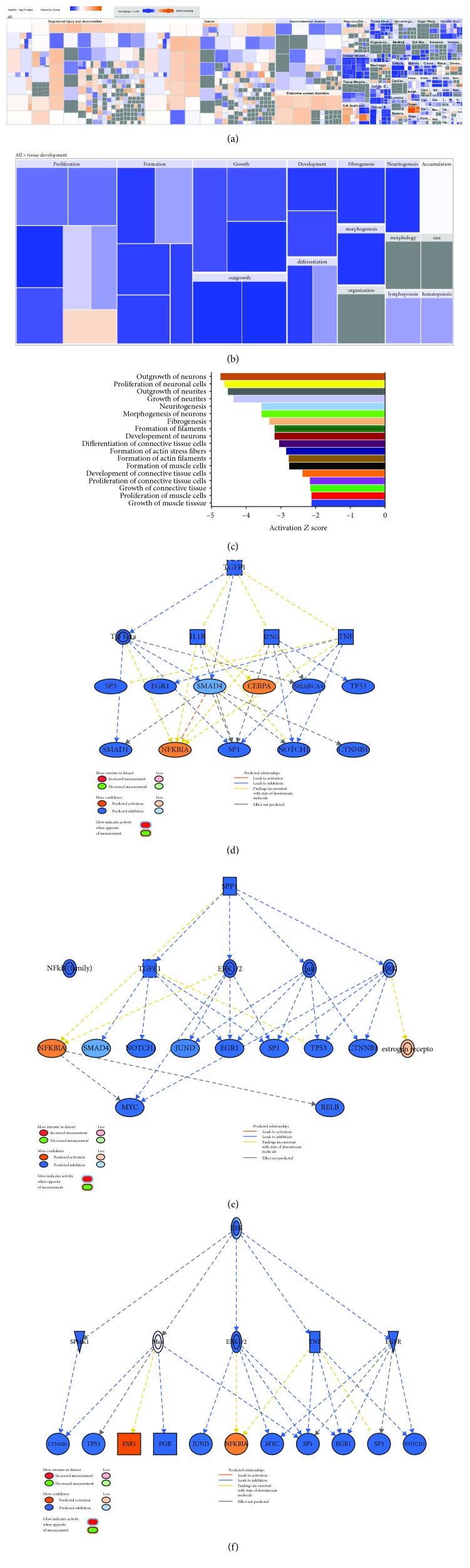
Inhibition of tissue development functional category and NOTCH-associated signaling networks in LY411575-treated hBMSCs. (a) Disease and function heat map depicting activation (red) or inhibition (blue) of the indicated functional and disease categories identified in the differentially expressed transcripts in LY411575-treated hBMSCs. (b, c) Heat map-illustrating affected tissue development functional category and associated functional annotations, respectively. Illustration of the TGF*β*1 (d), SPP1 (e), and ERK (f) genetic networks with predicted activated state of the network based on transcriptome data and with subsequent predicted effects on downstream effector molecules. Figure legend illustrates the relationship between molecules within the network.

We subsequently determined the enriched functional categories and intracellular signaling networks regulated by LY411575 during the osteogenic differentiation of hBMSCs. The list of differentially expressed genes was subjected to core significance analysis using manually curated human functional category annotations and network databases. Disease and functional analysis revealed a significant reduction in the gene expression in several functional categories including those involved in tissue development as illustrated in Figures 4(b) and 4(c). Upstream regulator analysis revealed several networks: TGF*β*, SPP1, and ERK, with suppressed upstream regulators and suppression of NOTCH activity (Figures 4(d)–4(f)). Our data suggest that LY411575 regulates a number of signaling network beyond Notch signaling to inhibit osteoblastic differentiation of hBMSCs.

### 3.3. LY411575 Inhibits *In Vivo* Ectopic Bone Formation

**Figure 5:**
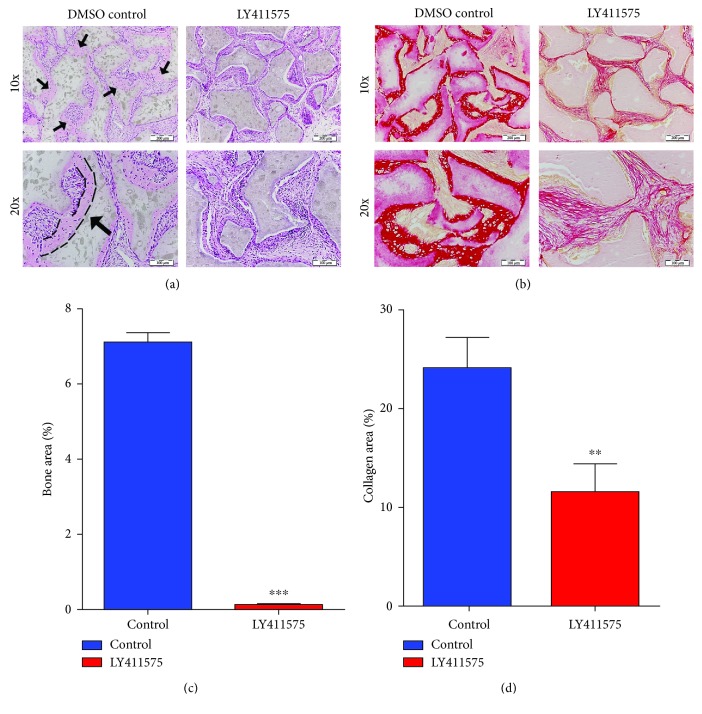
LY411575 inhibits *in vivo* ectopic bone formation. LY411575-treated hBMSCs and control hBMSCs were implanted with hydroxyl apatite/tricalcium phosphate (HA/TCP) subcutaneously into immune-deficient mice. The histology of in vivo bone formation was examined using H&E (a) and Sirius red (b) staining. Black arrows in (a) indicate bone formation (magnification 10x), and black line shows the bone formed zone (magnification 20x). In Sirius red-stained slides (b), red color indicates collagen tissue staining. Magnification 10x (first row; scale bar = 200 *μ*m) and magnification 20x (second row; scale bar = 100 *μ*m). Quantification of ectopic bone formation was performed with H&E (c) and Sirius red (d) staining (*n* = 3 implants/treatment) ^∗∗^*P* < 0.01; ^∗∗∗^*P* < 0.001. H&E: hematoxylin and eosin.

LY411575-treated hBMSCs compared to vehicle-treated control cells formed significantly lesser amount of ectopic bone following subcutaneous implantation in immune-deficient mice (Figures 5(a) and 5(b)) and quantitative histological analysis revealed >90% inhibition of bone area (Figure 5(c)) and >50% inhibition of collagen formation (Figure 5(d)).

## 4. Discussion

Bone marrow MSCs are recruited to bone formation sites during bone remodeling where they undergo differentiation into mature bone-forming osteoblastic cells [21]. Identifying the molecular mechanisms regulating osteoblast differentiation is relevant for understanding the pathogenesis of metabolic bone diseases and developing therapeutic targets for clinical intervention. Chemical biology approaches employing small molecule inhibitors that target specific intracellular signaling pathways have been exploited in recent years to identify the control mechanisms of stem cell differentiation and in drug discovery [11, 12, 22]. In the current study, we identified LY411575, during small molecule library functional screen, as a potent inhibitor of hBMSC osteoblast differentiation.

LY411575 is a cell-permeable *γ*-secretase inhibitor and thus is a potent inhibitor of Notch activation [23–25]. LY411575 was shown to block Notch activation *in vitro*, to induce apoptosis in Kaposi's sarcoma, to promote neural differentiation of mouse embryonic stem cells, and also to promote intestinal goblet cell differentiation in a mouse model of colitis [26–28].

We observed that LY411575 treatment impaired osteoblast differentiation *in vitro* and reduced ectopic bone formation *in vivo*. The role of Notch signaling in regulating osteoblast differentiation has been reported in several studies using primary human and murine MSCs [29–31], and these studies have reported that Notch signaling promoted osteoblast differentiation which is concordant with our data.

Employing global gene expression profiling of hBMSC following treatment with LY411575, we observed significant changes in multiple intracellular signaling pathways including focal adhesion, insulin, TGF*β*, and IL6, in addition to Notch signaling, suggesting that LY411575 interacts with multiple signaling pathways. It is plausible that LY411575 treatment leads to changes in a number of signaling pathways, secondary to its effects on Notch signaling, and suggests the existence of crosstalks between Notch signaling and other signaling pathways. For example, Zavadil and colleagues reported functional integration between Notch and TGF*β* signaling during epithelial to mesenchymal transition (EMT) [32]. Similar crosstalk between Notch and insulin signaling has been reported in lung adenocarcinoma cells under hypoxic environment [33]. Also, LY411575-treated hBMSCs exhibited significant downregulation (~-21.0 FC) of the ID3 gene expression which is one of the BMP-signaling target genes. Maeda et al. [34] have reported that ID1 and ID3 promoted bone formation in response to BMP stimulation in vivo and that ID1, ID2, and ID3 enhanced cell proliferation of early osteoblast progenitors [35]. Finally, previous published studies have demonstrated the importance of these signaling pathways in regulating osteoblasts differentiation including focal adhesion [36], insulin [37–39], TGF*β* [4, 40], IL6 [41, 42], Notch signaling [3, 9, 31], and cell cycle regulation [43].

Recently, Chen et al. [44] have reported that LY411575 suppressed osteoclast differentiation and bone resorption via suppressing Notch signaling. This study corroborates that Notch signaling in addition to its effects on osteoblast differentiation supports osteoclast functions and bone resorption. Thus, LY411575 can regulate both osteoblastic and osteoclastic activity and can be a good approach for treating bone diseases with mixed sclerotic and osteolytic lesions, e.g., cancer bone metastases.

## 5. Conclusions

LY411575 has been used for the treatment of a variety of neurological disorders including Alzheimer's disease and [45] has potential therapeutic use in the treatment of Kaposi's sarcoma and breast and colorectal cancer. Our data suggest the possible use of LY411575 in skeletal diseases associated with increased bone formation, e.g., osteosclerotic bone metastases or ectopic calcification association with renal osteodystrophy.

## Figures and Tables

**Table 1:** List of SYBR-Green primers used in current study.

Gene name	Forward primer	Reverse primer
ACTB	5′AGCCATGTACGTTGCTA	5′AGTCCGCCTAGAAGCA
ALPL	5′GGAACTCCTGACCCTTGACC3′	5′TCCTGTTCAGCTCGTACTGC3′
COL1A1	5′GAGTGCTGTCCCGTCTGC3′	5′TTTCTTGGTCGGTGGGTG3′
Osteonectin	5′GAGGAAACCGAAGAGGAGG3′	5′GGGGTGTTGTTCTCATCCAG3′
RUNX2	5′GTAGATGGACCTCGGGAACC3′	5′GAGGCGGTCAGAGAACAAAC3′
LIF	5′GCCACCCATGTCACAACAAC	5′CCCCCTGGGCTGTGTAATAG
NOTCH3	5′CCTGTGGCCCTCATGGTATC	5′CATGGGTTGGGGTCACAGTC
RRAD	5′GCGGAAACCCTAAAGTCCGA	5′GTCCGGGACCGTCCACT
IL6	CGAGCCCACCGGGAACGAAA	GGACCGAAGGCGTTGTGGAG
COL4A1	ATCCGGGTCTTCCTGGCCCC	CCGGTGTCACCACGACTGCC
ID3	5′TCATCTCCAACGACAAAAGG	5′ACCAGGTTTAGTCTCCAGGAA
TNF	5′ACTTTGGAGTGATCGGCC3′	5′GCTTGAGGGTTTGCTACAAC3′

## Data Availability

Supporting data are provided as supplementary data.

## Supplementary Materials

Supplementary Materials Supplementary Table 1: list of differentially expressed genes (2.0 FC, *P*(corr) < 0.05) in human bone marrow mesenchymal stem cells (hBMSCs) differentiated into osteoblasts (day 10) in the presence of LY411575 compared to DMSO.
